# Promoting Cardiac Regeneration and Repair Using Acellular Biomaterials

**DOI:** 10.3389/fbioe.2020.00291

**Published:** 2020-04-17

**Authors:** Vishnu Vasanthan, Ali Fatehi Hassanabad, Simranjit Pattar, Paul Niklewski, Karl Wagner, Paul W. M. Fedak

**Affiliations:** ^1^Section of Cardiac Surgery, Department of Cardiac Sciences, Libin Cardiovascular Institute, Cumming School of Medicine, University of Calgary, Calgary, AB, Canada; ^2^MDP Solutions, Cincinnati, OH, United States; ^3^Department of Pharmacology & Systems Physiology, College of Medicine, University of Cincinnati, Cincinnati, OH, United States; ^4^Health Economics and Clinical Outcomes Research, Xavier University, Cincinnati, OH, United States; ^5^Department of Chemical Engineering and Applied Chemistry, University of Toronto, Toronto, ON, Canada; ^6^Institute of Biomaterials and Biomedical Engineering, University of Toronto, Toronto, ON, Canada

**Keywords:** heart failure, biomaterials, cardiac regeneration, angiogenesis, fibrosis, cardiac surgery, ischemic heart disease

## Abstract

Ischemic heart disease is a common cause of end-stage heart failure and has persisted as one of the main causes of end stage heart failure requiring transplantation. Maladaptive myocardial remodeling due to ischemic injury involves multiple cell types and physiologic mechanisms. Pathogenic post-infarct remodeling involves collagen deposition, chamber dilatation and ventricular dysfunction. There have been significant improvements in medication and revascularization strategies. However, despite medical optimization and opportunities to restore blood flow, physicians lack therapies that directly access and manipulate the heart to promote healthy post-infarct myocardial remodeling. Strategies are now arising that use bioactive materials to promote cardiac regeneration by promoting angiogenesis and inhibiting cardiac fibrosis; and many of these strategies leverage the unique advantage of cardiac surgery to directly visualize and manipulate the heart. Although cellular-based strategies are emerging, multiple barriers exist for clinical translation. Acellular materials have also demonstrated preclinical therapeutic potential to promote angiogenesis and attenuate fibrosis and may be able to surmount these translational barriers. Within this review we outline various acellular biomaterials and we define epicardial infarct repair and intramyocardial injection, which focus on administering bioactive materials to the cardiac epicardium and myocardium respectively to promote cardiac regeneration. In conjunction with optimized medical therapy and revascularization, these techniques show promise to upregulate pathways of cardiac regeneration to preserve heart function.

## Introduction

### Heart Failure: Overview, Molecular Mechanisms and Gaps in Therapy

Ischemic heart disease is a common cause of end-stage heart failure that accounted for 38% of cardiac transplantation in the International Society of Heart and Lung Transplantation Database from 2005 to 2008, and has persisted as one of the main causes of end stage heart failure requiring transplantation from 1982 to 2012 (Taylor et al., 2009). Heart failure is a clinical syndrome of systemic malperfusion that affects 600,000 Canadians, with 50,000 new cases diagnosed each year; and has cost the Canadian healthcare system $2.8 billion per year (Heart and Stroke Foundation, 2016). Worldwide, heart failure affects 26 million people and accounts to 1-3% of healthcare expenditure in North America, Latin America, and Western Europe (Ambrosy et al., 2014; Ponikowski et al., 2014; Savarese and Lund, 2017).

In understanding ischemic heart disease, a brief review of the injurious cascade is beneficial. Pathologic myocardial remodeling due to ischemic injury involves multiple cell types and physiologic mechanisms. One key cell type is the cardiac fibroblast, an important modulator in both the structure and function of the heart. Playing a crucial role in extracellular matrix (ECM) regulation, cardiac fibroblasts modulate the rate of turnover and collagen deposition, both of which contribute to matrix dysregulation when upregulated. In an activated state, cardiac fibroblasts dysregulate the ECM by increasing ECM turnover via matrix metalloproteinase 9 (MMP-9) production and collagen deposition (Swynghedauw, 1999; Ducharme et al., 2000; Romanic et al., 2002; King et al., 2003; Fedak et al., 2005). MMPs are important players in ECM turnover release is upregulated in activated myofibroblasts; and they also reside in a pro-enzyme state in the extracellular matrix (Gallagher et al., 2007; Davis and Molkentin, 2014). Once release is upregulated by fibroblasts, they degrade connective proteins in the ECM like collagen; and degredation products of the ECM also have been found to stimulate new collagen deposition (Gallagher et al., 2007). Ultimately, this pathway is one of the keys to pathologic ventricular remodeling as it contributes to breakdown of healthy ECM and malapdaptive collagen deposition.

The Renin-Angiotensin-Aldosterone System (RAAS) has been highlighted as a key mechanism that is activated by chamber distension. Increased levels of Angiotensin II promote cardiac fibroblast activation. Ultimately, this sequence results in a profibrotic state linked to eccentric hypertrophy, chamber dilatation and congestive heart failure. Neurohormonal adrenergic pathways may serve as short term compensation, but chronically upregulate RAAS and therefore fibroblast activation. Inflammatory cytokines such as TNF-α are overexpressed during heart failure and have been linked with increased MMP levels, contributing to ECM dysregulation and heart failure (Mann, 2002; Fedak et al., 2005; Hartupee and Mann, 2017).

Along with providing an initial insult that initiates the aforementioned pathways, ischemic injury results in endothelial injury and cardiomyocyte death, which induces the release of a variety of inflammatory cytokines including TNF-α, IL-1a, IL-1b, IL-6, and IL-8 (Kawaguchi et al., 2011; Shetelig et al., 2018; Pattar et al., 2019a). At this point, immune cells are also recruited and contribute to further upregulation of inflammatory signaling and upregulation of proinflammatory and profibrotic cytokine TGF-β (Frangogiannis, 2006; Shinde and Frangogiannis, 2014). Among other cytokines, TBG-β promotes fibroblast activation into myofibroblasts, which dysregulate the extracellular matrix. In their activated state, myofibroblasts exhibit a pro-fibrotic phenotype and ECM dysregulation eventually results in increased collagen deposition (Brown et al., 2005; Travers et al., 2016; Pattar et al., 2019a). Though collagen deposition initially serves benefit by strengthening the ventricular wall to prevent infarction, the benefit is overshadowed by decreased contractility of the ventricle and impairment of ventricular relaxation. Ultimately, if the size of the infarct is large enough, this pathway causes ventricular dilatation as well as systolic and diastolic dysfunction which result in congestive heart failure. Coronary artery disease and subsequent ischemic injury are inciting events that cause ventricular dilatation and dysfunction (Briceno et al., 2016). Regarding ischemic heart disease and subsequent heart failure, there have been significant improvements in medication and revascularization strategies (Hillis et al., 2011; Levine et al., 2011; O’Gara et al., 2013; Fihn et al., 2014). However, despite medical optimization and opportunities to restore blood flow, physicians lack therapies that directly access and manipulate the heart to promote healthy post-infarct myocardial remodeling (Mewhort et al., 2014, 2017; Svystonyuk et al., 2018; McLaughlin et al., 2019).

### Acellular Materials Capable of Cardiac Repair

In recent years, acellular biomaterials have demonstrated potential for cardiac regeneration by promoting proangiogenic and antifibrotic pathways. Broadly, acellular biomaterials used in cardiac therapies are either derived from tissue sources or synthesized artificially. Though cellular therapies are gaining popularity and demonstrate promise in preclinical studies, clinical translation is accompanied with difficulties involving clinical effectiveness and feasibility (Balsam et al., 2004; Murry et al., 2004; Gyongyosi et al., 2015; Kaiser, 2018; Pattar et al., 2019a). Acellular materials have demonstrated preclinical therapeutic potential due to multiple proposed mechanisms including but not limited to growth factor release and restoration of key components of extracellular matrix (Mewhort et al., 2017; McLaughlin et al., 2019; Pattar et al., 2019a, b). As well, many acellular materials have already received approval from regulatory bodies like the Food and Drug Administration and Health Canada, which possibly decreases barriers to clinical translation.

Several commercially available patch biomaterials are composed of decellularized extracellular matrix (ECM) from multiple tissue sources. Numerous decellularization techniques are outlined in the literature; but they can be categorized as chemical (acid-base, hypotonic-hypertonic, detergents, alcohol), biologic (collagenase, trypsin, nuclease, ethylenediaminetriacetic acid), physical (temperature, mechanical abrasion, pressure, electroporation) and combinations of the aforementioned (Crapo et al., 2011; Gilpin and Yang, 2017). Examples include glutaraldehyde-fixed bovine pericardium which is commonly used for structural support in cardiac surgery; and unfixed small intestinal submucosa extracellular matrix (SIS-ECM) which is commonly investigated for potential bioactive effects (Mewhort et al., 2017). ECM, especially collagen, is well preserved across species so acellular components usually do not cause significant immune reaction (Saldin et al., 2017; Copes et al., 2019). ECM-based biomaterials are composed mainly of collagen and a variety of glycosaminoglycans including hyaluronic acid, chondroitin sulfate, heparin sulfate (Saldin et al., 2017). Despite being devoid of resident fibroblasts among other cell populations, decellularized ECM-based biomaterials retain various growth factors including FGF-2, VEGF and HGF, which may confer regenerative effects (Mewhort et al., 2014, 2017; Svystonyuk et al., 2018; Copes et al., 2019). Other synthetic materials are also used in cardiac surgery mainly for structural purposes like GORE-TEX^®^, polytetrafluoroethylene, polyester-urethane, and textile grafts, but they will not be discussed in this review because they are not prepared from extracellular matrix components nor are they loaded with growth factors during manufacturing.

Other than patch-based materials, hydrogels also show promise for cardiac regeneration. Like patch-based materials, they can be derived from multiple sources including but not limited to the extracellular matrix, recombinant collagen, proteoglycans, alginates, and synthetic polymers (Liu et al., 2002; Toeg et al., 2013; Mann et al., 2016; Spang and Christman, 2018; Matsumura et al., 2019; McLaughlin et al., 2019). These materials can also contain growth factors depending on their composition (Hao et al., 2007; Toeg et al., 2013). Hydrogels cannot be used for reconstruction in the same way as patch materials, but regeneration via intramyocardial injection is thought to occur through providing growth factors or replenishing damaged proteins (Hao et al., 2007; Jourdan-LeSaux et al., 2010; Maeda and Ruel, 2015; McLaughlin et al., 2019).

Herein, we define and review promising surgical strategies that use acellular biomaterials to augment myocardial remodeling by promoting a pro-angiogenic and antifibrotic environment for improved post-infarct cardiac remodeling. We will focus on Epicardial Infarct Repair (EIR) and Intramyocardial Injection (IMI) because they are both procedures that can be used in conjunction with coronary artery bypass surgery to further improve cardiac function after revascularization. Additionally, with new materials entering this growing space, we will review preclinical evaluation modalities used to assess promising biomaterials for EIR and IMI.

## Patch Biomaterials: Overview, Preparation Strategies and Complications

### Overview

Epicardial infarct repair is an emerging surgical technique that involves placing bioactive patch materials on the epicardium via suture to modulate pathways toward regeneration (Mewhort et al., 2014, 2016, 2017). This method takes advantage of a cardiac surgeon’s unique ability to readily access the epicardium, a thin mesothelial layer containing multiple cell populations. When the patch is sewn on the heart, it both makes direct contact with the epicardium and, depending on the material, can release growth factors. Direct contact and growth factor release may both play a role in modulating cell populations to promote angiogenesis and attenuate fibrosis (Mewhort et al., 2016, 2017; Svystonyuk et al., 2018; Pattar et al., 2019a).

**FIGURE 1:**
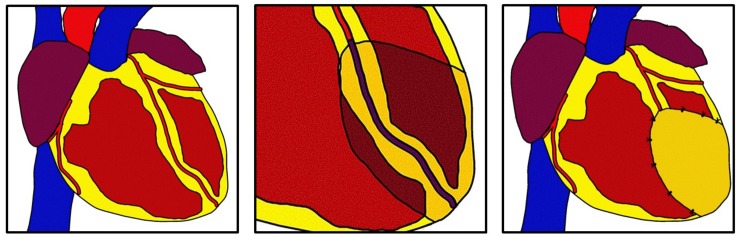
Epicardial infarct repair. After myocardial infarction a bioinductive patch is sewn using simple interrupted sutures over the infarcted area. The patch modulates epicardial and myocardial cells to promote angiogenesis and antifibrosis through direct contact or elution of growth factors. This technique can be used in concomitance with revascularization.

Epicardial derived progenitor cells (EPDCs) may be key players in EIR-mediated regeneration because they can undergo epithelial to mesenchymal transition (EMT) to differentiate into myocardial cell types including fibroblasts, vascular smooth muscle cells, and possibly cardiomyocytes and endothelial cells (Smart and Riley, 2012). Thus, modulation of pathways may promote differentiation to replace those lost to ischemic events. Additionally, growth factors such as VEGF and FGF-2 may act on underlying fibroblasts to attenuate adverse remodeling (Mewhort et al., 2014, 2017; Svystonyuk et al., 2015). Figure 1 depicts general concepts of EIR.

### Unfixed Biomaterials

Acellular porcine small intestinal submucosal ECM (SIS-ECM) has been the most common unfixed biomaterial used in cardiac surgery, and it is one of the most extensively examined for use in cardiac regeneration. *In vitro*, acellular porcine SIS-ECM has promoted a quiescent, pro-angiogenic and antifibrotic fibroblast phenotype. The main pathway is thought to be through FGF-2 release, which inhibits pro-fibrotic TGF-B pathways (Svystonyuk et al., 2015; Mewhort et al., 2017). Previous studies have shown that FGF-2 and VEGF eluted from these materials confer antifibrotic effects by both inhibiting fibroblast activation into myofibroblasts and increasing fibroblast production of additional FGF-2 (Mewhort et al., 2014, 2017; Svystonyuk et al., 2018; Pattar et al., 2019a, b). In rat myocardial infarct models, echocardiography findings showed improvements in cardiac function; and histology showed CorMatrix has demonstrated increases in neoangiogenesis in the infarct and peri-infarct regions (Mewhort et al., 2017). Porcine models showed increased rest myocardial perfusion on MRI and increased wall thickening in areas treated by the patch (Mewhort et al., 2016). Additionally, it has been shown in preclinical models that diastolic relaxation, representative or left ventricular compliance, is not impeded with the application of 4-ply lyophilized SIS-ECM (Mewhort et al., 2016). Having been used in pre-clinical models, current data has served as the basis for clinical trials using the commercially available porcine SIS-ECM, CorMatrix (CorMatrix Cardiovascular Inc., United States; NCT02887768).

### Fixed Biomaterials

Glutaraldehyde fixation is a common strategy to prepare biomaterials in cardiac surgery but has not demonstrated promise for EIR in preclinical evaluation. Crosslinking caused by glutaraldehyde increases biomaterial tensile strength, allowing it to be useful in cardiac reconstruction. Glutaraldehyde materials, typically based from xenogenic or autologous pericardium, are commonly used in reconstruction of multiple structures in the heart including septal defects/ruptures between atria and ventricles, along with reconstruction of areas of the heart obliterated by infective endocarditis (David et al., 1995; David and Armstrong, 1998; De La Zerda et al., 2008; Bedeir et al., 2014; Ozaki et al., 2018; Hiromoto et al., 2019). For decades, glutaraldehyde fixed bovine pericardium has been used in bioprosthetic heart valve replacements (Wallace, 1975; Schoen and Levy, 2005; Manji et al., 2006; Mirzaie et al., 2007). Preclinical studies have shown that glutaraldehyde fixed porcine SIS-ECM did not promote a proangiogenic or antifibrotic pathway when fibroblasts were seeded, and glutaraldehyde fixed SIS-ECM also did not confer angiogenic or antifibrotic effects in rat infarct models when compared to unfixed SIS-ECM, suggesting that glutaraldehyde in part neutralizes or decreases the biogenic effects of SIS-ECM (Mewhort et al., 2017). Rat models treated with EIR using glutaraldyhyde-fixed SIS-ECM did not demonstrate improvements in pressure-volume loops, echocardiography, intramyocardial FGF-2 or angiogenesis-focused histology (Mewhort et al., 2017).

Dye-mediated oxidation, or photofixation, is another form of fixation that does not primarily rely on glutaraldehyde. Pre-clinical *in vivo* models, focusing mainly on vascular and cardiac reconstruction have demonstrated that photofixed bovine pericardium is less immunogenic and calcific when compared to materials made from glutaraldehyde fixation (Bianco et al., 1996; Moore and Phillips, 1997; Moore et al., 1998; Pattar et al., 2019a). Materials developed using photo-oxidation have been used in multiple forms of cardiac and vascular reconstruction. Cases have shown congenital reconstruction of the right ventricular outflow tract, pulmonary artery, septal defects, superior vena cava reconstruction, aortic arch procedures and valvular reconstruction with minimal immune-related rejection (Majeed et al., 2016; Baird et al., 2017). However, they have not been reported in the context of EIR.

### Complications

Small intestinal submucosal patches have been used for multiple clinical purposes in cardiac surgery. Uses include ventricular septal repair, atrial septal defect repair, aortic root enlargement, mitral valve repair, tricuspid valve repair, repairs of structural complications of endocarditis and pediatric cardiac surgery (Witt et al., 2013; Yanagawa et al., 2013; Brinster and Patel, 2014; Gerdisch et al., 2014; Sundermann et al., 2014). Though relatively well tolerated in adults, in pediatric cases it was found to cause an immune reaction resulting in localized inflammation in the pediatric complication (Rosario-Quinones et al., 2015; Woo et al., 2016).

Calcification of glutaraldehyde-fixed tissue implants has been one of their most significant issues since their introduction in the 1960s because of subsequent implant calcification (Schoen et al., 1986; Liu et al., 2014). While the exact mechanism of aldehyde-related calcification is not known, it is thought to be due to a high degree of crosslinking, unreacted aldehyde groups and their attraction of calcium ions, disruption of cellular calcium regulation, localization of calcium in areas of stress and presence of lipids and cell debris (Schmidt and Baier, 2000). Attempts at reducing calcification seem to focus on eliminating cell remnants and lipids and have demonstrated varied success (Arbustini et al., 1984; Chen et al., 1994; Gott et al., 1997; Liu et al., 2014). Though not used in EIR, the use of glutaraldehyde-fixed products in bioprosthetic valves eventually results in calcification (Golomb et al., 1987; Schoen et al., 1992; Girardot et al., 1995; Bonetti et al., 2019).

Photo-fixation has been used as another attempt to minimize calcification. Bovine pericardium fixed via dye-mediated oxidation, or photo-fixation has been used in cardiovascular surgery in pediatric patients. Though photo-fixation has demonstrated accelerated degeneration compared to glutaraldehyde fixed autologous pericardium, these patches seemed to have better rates of degeneration when compared to glutaraldehyde fixed bovine pericardium (Majeed et al., 2016). Photo-fixated patches have demonstrated minimal complication rates due to severe calcification (0.3%, 1 of 317 pediatric patients) (Baird et al., 2017).

Despite these complications being listed, the aforementioned biomaterials were used in the context of reconstruction and providing functional support. On the other hand, their use in EIR is a vehicle for cardiac regeneration through bioinductive properties. The most relevant complications from previous literature are likely calcification and localized inflammation, which could affect the pericardial space ventricular movement when used in EIR.

## Intramyocardial Injections: Overview, Preparations and Complications

### Overview

**FIGURE 2:**
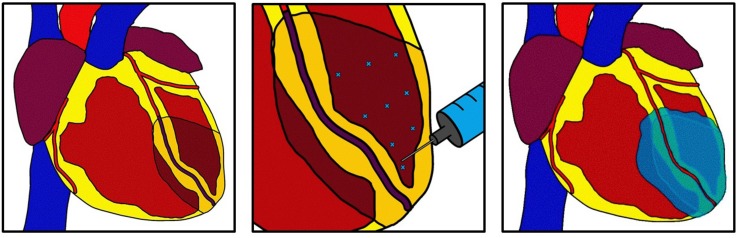
Intramyocardial injection. After myocardial infarction a hydrogel derived from multiple possible sources is injected into the superficial myocardial using multiple injections. The hydrogel can distribute beyond the infarct zone and into the border zone. The hydrogel can act as a bulking agent to restore myocardial structure or can deliver growth factors to targeted areas. This technique can be used in concomitance with revascularization.

Intramyocardial Injection (IMI) involves injecting hydrogels into the superficial myocardium to promote neoangiogenesis and attenuate pathologic remodeling (Hao et al., 2007; Toeg et al., 2013; McLaughlin et al., 2019). Figure 2 depicts basic concepts of intramyocardial injection. Using an injectable approach still takes advantage of a surgeon’s ability to directly access the heart; and this method precludes the need for suturing which may increase compatibility with minimally invasive surgery (Maeda and Ruel, 2015; McLaughlin et al., 2019). These materials are thought to promote neovascularization and attenuate fibrosis by providing growth factors and replenishing ECM proteins or in infarcted areas. Cellular therapies exist for this technique that may leverage the benefits of target cell exosomes to trigger endogenous repair mechanisms (Amado et al., 2005; Karantalis et al., 2014). However, this section will focus on acellular therapies including injections of animal-derived ECM, recombinant human proteins and other hydrogels containing growth factors.

### Preparations of Injectable Hydrogels

Hydrogels are polymer-based networks that attract, contain and become swollen with water (Hoffman, 2002; Nguyen and Lee, 2010; Tous et al., 2011). They display tissue like qualities, but their key advantage for IMI is the ability to first act as a liquid and distribute into incumbent tissue and then self-assemble into a more semi-rigid structure. Depending on the preparation strategy, hydrogels have been used as myocardial bulking agents, vehicles to deliver therapy or a combination of both (Tous et al., 2011). Multiple preparation strategies exist, with ample pre-clinical evidence and a few in-human studies. Base materials range from animal-derived ECM, recombinant human proteins and synthetic materials (Liu et al., 2002; Hao et al., 2007; Deng et al., 2010; Toeg et al., 2013; Mann et al., 2016; Matsumura et al., 2019; McLaughlin et al., 2019). Self-assembly, the key feature of hydrogels, can be achieved through multiple means including non-covalent ionic interactions, covalent crosslinking and thermal transitions (Hoffman, 2002; Nguyen and Lee, 2010; Tous et al., 2011).

Injectable ECM-based hydrogels have gained attention for IMI. These hydrogels incorporate micronized versions of acellular unfixed ECM materials used in EIR, which allows injection into the myocardium of different ECM components including collagen, proteoglycans and residing growth factors. Preclinically, it has been demonstrated in a mouse model that comminuted porcine SIS-ECM can be injected into a myocardial infarction model and both improve ejection fraction on echocardiography; also it reduced infarct size and upregulate neovascularization as demonstrated on histology (Toeg et al., 2013). This study did not use a crosslinking agent, relying on non-covalent interactions; and the results showed comparable results between the acellular SIS-ECM group and a group that used a combination of SIS-ECM and circulating angiogenic cells (Toeg et al., 2013).

Studies have demonstrated the potential of unfixed xenogenic biomaterials to cause adverse immune reactions in patients, which may be due to animal-based proteins like endotoxins (Charriere et al., 1989; Badylak and Gilbert, 2008; Rosario-Quinones et al., 2015; Woo et al., 2016; McLaughlin et al., 2019). Rather than using animal-derived ECM, another option is human recombinant proteins found in ECM. In mice models, recombinant human collagen I and III matrices added to chondroitin sulfate-based hydrogels and crosslinked with *N*-ethyl-*N*-(3-dimethylaminopropyl) carbodiimide and *N*-hydroxysuccinimide have promoted neovascularization and attenuated fibrosis in the border zones of infarcted areas (McLaughlin et al., 2019). Given the disruption of normal ECM due to inflammatory cascades upregulating fibroblast activation in infarcted areas, it is thought that the re-introduction of normal connective tissue proteins like collagen can restore ECM and possibly have a 3-dimensional structural effect that allows for non-pathologic activity to resume (Jourdan-LeSaux et al., 2010; McLaughlin et al., 2019). Additionally, this strategy promotes M2 macrophage differentiation and downregulates expression of MMP1 mRNA from macrophages, which may be supportive of cardiac regeneration (McLaughlin et al., 2019). Acellular therapy with human recombinant molecules is a promising and relatively new technique.

Hydrogels made from proteoglycans or artificial sources have also been shown to effectively house growth factors for delivery through IMI. Proteoglycans such as hyaluronan and chitosan have also been used to make hydrogels for IMI. The net negative charge can attract and hold positively charged growth factors, enabling slow delivery once injected into the myocardium (Yun et al., 2010). Artificial hydrogels were also made with alginates or polyethylene glycol and were shown to house growth factors for administration. In rats, an FGF-2 enriched artificial crosslinked hydrogel made from *N*-isopropylacrylamide (NIPAAm), 2-Hydroxylethyl methacrylate (HEMA) and dextran improved neovascularization, decreases collagen deposition and improved cardiac function (Zhu H. et al., 2017). Other preclinical studies show promising results for artificial hydrogels using non-animal and non-human hydrogels, with better neovascularization and tensile strength in rats and decreased scar on MRI in pigs (Matsumura et al., 2019). The AUGMENT HF human trial (NCT01311791) used an alginate-based gel and showed short term improvements in ejection fraction, left ventricular end diastolic diameter and left ventricular end systolic function; but there were no long-term improvements (Mann et al., 2016; Zhu Y. et al., 2017).

### Complications

Unlike with patch biomaterials, hydrogels used for IMI were initially made with this purpose; so complications found in the literature are more relevant. Overall, a suspected complication was mucosal oozing at injection sites when used in patients with heart failure, especially those undergoing ventricular assist device insertion where this complication is more common. However, it was found that the incidence of this complication was not increased in the limited cases performed, though it should be noted this study used a cell-based therapy (Yau et al., 2019). Given IMI involves injecting a foreign substance directly into the myocardium, a theoretical risk could be if the gel ruptures through the endocardium and embolizes, causing neurological and systemic complications. However, with limited clinical evidence available, catastrophic embolism-related events like stroke were not increased with IMI in the AUGMENT-HF trial using an alginate-based hydrogel (Mann et al., 2016). Overall, intramyocardial injection seems to be a low-complication procedure; but there remains a paucity of long-term data for this therapy.

## Evaluation of Biomaterials

As new biomaterials continue to emerge with potential for EIR or IMI, a set of reproducible evaluation modalities will be required for effective comparison. Table 1 briefly summarizes relevant evaluation modalities which are further elaborated below.

**TABLE 1:** Preclinical evaluation modalities for biomaterials.

**Test**	**Brief description**	**Relevant findings**
Scanning Electron Microscopy	Assessment of pore size distribution and subjective evaluation of biomaterials	Subjective evaluation of patch surfaces porosity of hydrogels
Scanning Calorimeter	Determine endothermic peaks which correlate with denaturation temperatures of hydrogels	Denaturation temperature
Hydrogel water content	Determining wet and dry weight of hydrogel to calculate percent water content	Wet and dry weights to calculate water content
Radiolabeled hydrogel tracking	Using fluorescent tag, trace the distribution of the hydrogel after injection and assess how long it stays present post-injection	Extent of distribution of hydrogels across the epicardium and duration of time taken for degradation
Young’s Modulus for Biomaterial	Tensile strength of patch assessed using Young’s Modulus for strain	Maximum strain tolerated by biomaterial
Elution studies	Biomaterials submerged in prespecified volume of medium, typically serum-free cell culture media. After specified time, growth factors of conditioned media measured with multiplex assay	Concentrations of growth factors, which can be compared to a serum free control
Growth Factor Quantification after Seeding Fibroblasts	Cardiac fibroblasts seeded onto biomaterials in serum-free culture media. Conditioned media collected after specified time, growth factors quantified via multiplex assay	Concentrations of growth factors, which can be compared to serum free control without biomaterials
HUVEC Angiogenesis Assay	HUVEC cells are added to a protein-based gel matrix. Groups are exposed to various biomaterials which can be floated in cell culture media. After a pre-specified timepoint, light microscopy can visualize cell networks and networks can be analyzed with imaging software	Length of tubules, number of nodes and junctions in the network
Echocardiography	For large and small animal models. Assessment of cardiac structure and function in response to different therapies. Important measurements include EF, fractional contraction, LVESD, LVEDD, Wall thickness	Ejection fraction, fractional area contraction, systolic and diastolic ventricular size, abnormalities in wall motion
Magnetic Resonance Imaging	For large animal models. Assessment of cardiac structure, function, perfusion and scar formation. Important measurements include EF, fractional contraction, LVESD, LVEDD, Wall thickness, perfusion assessments and late gadolinium enhancement	Chamber size and function, scar volume, myocardial perfusion
Histology and Immunohistochemistry	For Large or Small Animal Models. Multiple staining techniques. Can visualize scar deposition, viable cardiomyocytes, blood vessel density, ventricular wall thickness	Quantification of blood vessels in view, proportion of scar, myocyte viability
Young Modulus for Myocardium	Changes in tensile strength of ventricular myocardium after induced infarction and subsequent treatment with various therapies	Maximum strain tolerated by animal ventricular myocardium

### Evaluation of Biomaterial Physical Characteristics

Prior to assessing bioactive components, the physical properties of biomaterials can be examined. Uniaxial tensile tests include pulling specimens apart until failure can be used to assess Young’s modulus and tensile strength patch biomaterials (Neethling et al., 2018). Tensile strength can also be used to assess robustness for suturing. Scanning electron microscopy can be used to look at the surface and porosity of biomaterials to help predict how incumbent cells will infiltrate the material (McLaughlin et al., 2019). With the possibility of constant or repetitive force being placed on these biomaterials, viscoelastic moduli may help characterize mechanical responses to assess stress-strain relationships of biomaterials over time (Huang et al., 2019). Hydrogels used for IMI can also be assessed for denaturation temperatures, gelation time, degree of cross linking, viscosity and water content (Pena et al., 2018; McLaughlin et al., 2019).

### *In vitro* and *ex vivo* Assessment

Many of the aforementioned biomaterials present the opportunity for evaluation of growth factors before and after seeding of cells. With multiple biomaterials acting through a paracrine mechanism, elution studies can be performed to determine growth factors released into a pre-specified media. Comparisons can be made between different biomaterials. Additionally, fibroblasts can be seeded onto biomaterials to assess exosomal modification caused by different biomaterials when compared to control; and growth factors can be measured to see how fibroblast interaction with the material changes growth factor production (Mewhort et al., 2017).

Though not as well established, different *in vitro* assays have been used to demonstrate angiogenic potential of biomaterials. Previously, human umbilical vein endothelial cells (HUVECs) were seeded to patch biomaterials and their arrangements were measured using imaging software; and those seeded onto bioactive materials demonstrated increased arrangement parameters representative of angiogenesis (Mewhort et al., 2017). Angiogenic potential of hydrogels for IMI can be analyzed via surface seeding of endothelial cells followed by quantification of structural sprouting and endothelial cell dispersion within a 3D matrix (Chen and Kaji, 2017).

With more sophisticated cell culture techniques continuing to develop, microdevices are providing new opportunities for *ex vivo* assessments of cardiac biomaterials (Osaki et al., 2018). In an attempt to emulate the physiology and complex interactions present in native human tissues, the “organ-on-a-chip” helps advance *in vitro* tissue culture (Zhang and Radisic, 2017). Tissue-on-a-chip models combine cells and biomaterials on microfabricated platforms to simulate organ structures and functions in a controlled and accessible environment (Ahadian et al., 2018). Co-culturing multiple cell types in 3D arrangements can generate more mechanistic insight toward predicting *in vivo* efficacy than 2D cell cultures (Zhang and Radisic, 2017). Microfluidic vascularization is also possible in these models, enabling assessment of neovascularization (Zhang et al., 2016; Lai et al., 2017). These models are currently being developed to simulate ischemic injury to enable detailed investigation into the mechanisms of ischemic heart damage and the effects of therapeutic biomaterials at a mechanistic level (Chen and Vunjak-Novakovic, 2018). Larger scale milli-fluidic bioreactors use a larger scale device in comparison to microfluidic devices in order to facilitate live imaging of an artificially perfused environment while also using impedence- and resistance-based techniques to examine integrity of a layer of cells (Cacopardo et al., 2019).

### *In vivo* Assessment

*In vivo* models have also been used to evaluate biomaterials in both EIR and IMI. Rat infarct models have been used to assess cardiac function after addition of biomaterials using echocardiography, which can measure ejection fraction along with left ventricular end diastolic diameter, end systolic diameter and estimate LV mass (Mewhort et al., 2017). Porcine large animal models can also be used for functional assessment, providing a model that is more anatomically and physiologically similar to a human and also compatible with cardiac MRI (Mewhort et al., 2016). Both models can be used for histology to assess neorevascularization by counting the number of blood vessels in the visualized field (Mewhort et al., 2016, 2017). Studies to evaluate IMI using cellular and acellular therapeutics have also used multiple *in vivo* models, including mouse, rat and pig models; and these models facilitated evaluation by echocardiography and pressure volume loops, with the porcine models being compatible with MRI (Min et al., 2003; Klopsch et al., 2009; McCall et al., 2012; Toeg et al., 2013; Li et al., 2017; McLaughlin et al., 2019). MRI also allows for myocardial perfusion scans that may be able to better examine neorevascularization into the peri-infarct and infarcted region. Late gadolinium enhancement is a valuable aspect of cardiac MRI that allows investigators to identify scar in the myocardium and compare changes in viable myocardium and scar masses between different treatment groups (McCall et al., 2012; Mewhort et al., 2016).

Histology and immunohistochemistry for neovascularization, scar deposition and presence of cardiomyocytes can be performed post-mortem (Mewhort et al., 2016, 2017; McLaughlin et al., 2019). Given that IMI deals with an injectable gel, fluorescent labeling techniques can be used to trace injected materials to see how they traverse the epicardium and myocardium at timepoints post-application (McLaughlin et al., 2019).

### Proposed Ideal Characteristics of Acellular Biomaterials

Given the early stages of both of these procedures, ideal characteristics of materials for both EIR and IMI can only be hypothesized. For both procedures, it would inherently be preferred to use bioactive materials that are able to modulate cellular activity and exosomes on order to recruit cells to promote regeneration. Additionally, given that many patients with end-stage heart failure receive subsequent procedures including transplant and ventricular assist device implantation, non-immunogenic and non-calcific materials may be beneficial to mitigate formation of intrapericardial adhesions, which would make redo surgery technically challenging and higher risk. In EIR, it would be preferable that materials do not impede diastolic relaxation, thus a balance between tensile strength and elasticity may be key. In IMI, viscosity may be important characteristic to examine because ideal hydrogels would be able to permeate from the multiple microinjection sites to cover the infarct zone and border zone but still be able to take a semisolid form for structural support. As well, rate of degradation may require consideration as more ideal hydrogels will be retained in the myocardium over the course of post-infarct healing.

## Discussion

Bioactive materials that promote post-infarct cardiac regeneration present a game-changing opportunity to the field of cardiac sciences. Along with revascularization and medical management, surgeons can further guide healing by leveraging the unique opportunity to directly manipulate the heart. Reproducible evaluation strategies such as those discussed above will be needed for comparison, new pathways will continue to need to be explored and augmentation strategies may further enhance regeneration.

Though EIR and IMI have both been developed to promote cardiac regeneration, there are key differences between these approaches. As EIR places a patch on the epicardial surface while IMI directly introduces a hydrogel that redistributes in cardiac tissue, these techniques demonstrate different levels of incorporation of biomaterials (Nguyen and Lee, 2010; Tous et al., 2011; McCall et al., 2012; Mewhort et al., 2014, 2016, 2017; Zhu Y. et al., 2017; Svystonyuk et al., 2018; Pattar et al., 2019a). No direct comparisons have been performed but EIR may provide the benefit of external support on ischemic cardiac tissue, while IMI may provide benefit by improving structural integrity by immersion of hydrogels into the myocardium (Tous et al., 2011; Mewhort et al., 2017; Pattar et al., 2019a). EIR also may provide additional benefit as direct contact with the epicardium may improve recruitment of EPDC cells (Mewhort et al., 2017; Pattar et al., 2019a). However hydrogels in IMI allow for release of growth factors and possible replenishment of ECM components straight into the myocardium (Tous et al., 2011; Toeg et al., 2013; Matsumura et al., 2019; McLaughlin et al., 2019). Hydrogels degrade over time after IMI while biomaterial patches may last longer or resist degradation altogether, and it is currently unclear how these aspects may provide benefit or consequence. Degradation may allow hydrogels to provide bioactive effects and then degrade after they confer regenerative effects but long-term retention of materials in EIR may allow for long-term support of the ventricle (Hoffman, 2002; Tous et al., 2011; Mewhort et al., 2016, 2017; Pattar et al., 2019a). On the other hand, retention of foreign material may increase likelihood of inflammation or calcification (Manji et al., 2006; Witt et al., 2013; Rosario-Quinones et al., 2015; Neethling et al., 2018).

In terms of safety, initial preclinical experience in these techniques suggests they are both safe when performed appropriately. Current pre-clinical studies do not report complications related to sewing bioactive patches to the epicardium and there is currently a paucity of long-term data regarding mortality and stroke (Mewhort et al., 2016, 2017). As well, no long-term data currently exists in EIR to examine calcification or inflammation of bioactive patches as seen when SIS-ECM is used in select cases of cardiac reconstruction (Rosario-Quinones et al., 2015; Woo et al., 2016). In IMI, current data does not suggest any worsening of mortality or catastrophic embolism-related complications form the hydrogel rupturing into circulation (Mann et al., 2016).

Multiple future directions exist in this field. As the field advances, it may be beneficial to examine other pathways through which biomaterials promote regeneration. Given that the RAAS and adrenergic neurohormonal pathways are known key pathways involved in heart failure, identifying ways to further manipulate them using biomaterials may enhance EIR and IMI (Fedak et al., 2005). Augmentation of biomaterials may be key in order to enhance their regenerative effects and, ultimately, clinical benefit. Non-covalent augmentation by heparin may be a possible way to enhance growth factor loading capacity (Park et al., 2018). Additionally, growth factors like FGF-2 could possibly be loaded to biomaterials to enhance them (Mewhort et al., 2014).

Future uses of biomaterials for cardiac regeneration may extend beyond ischemic heart disease. It may be possible to apply these materials to cardiomyopathy unrelated to ischemic injury, to mitigate fibrosis and promote neoangiogenesis into cardiomyopathic areas. Thus, EIR and IMI may be useful to complement ventricular assist device insertion. As VADs decompress the heart and revascularization strategies will restore blood flow, application of bioactive materials in EIR and IMI will improve neoangiogenesis and antagonize fibrosis in more optimized conditions. With the waitlist mortality for cardiac transplantation increasing, cardiac regeneration therapies may help improve ventricular assist devices as destination therapy or bridge-to-recovery in patients, rather than bridge-to-transplantation (Lund et al., 2013). With further establishment in the clinical setting, the use of these materials may expand to treat other non-ischemic etiologies of heart failure and even improve cardiac remodeling after other cardiac procedures.

## Author Contributions

All authors listed have made a substantial, direct and intellectual contribution to the work, and approved it for publication.

## Conflict of Interest

PF is a consultant for CorMatrix Cardiovascular Inc. PN is a partner with MDP Solutions. The remaining authors declare that the research was conducted in the absence of any commercial or financial relationships that could be construed as a potential conflict of interest.
